# Biosurfactants and Their Applications in the Oil and Gas Industry: Current State of Knowledge and Future Perspectives

**DOI:** 10.3389/fbioe.2021.626639

**Published:** 2021-02-15

**Authors:** Christina Nikolova, Tony Gutierrez

**Affiliations:** School of Engineering and Physical Sciences, Institute of Mechanical, Process and Energy Engineering, Heriot-Watt University, Edinburgh, United Kingdom

**Keywords:** biosurfactants, surface-active agents, microbial enhanced oil recovery (MEOR), microorganisms, marine environment

## Abstract

Surfactants are a group of amphiphilic chemical compounds (i.e., having both hydrophobic and hydrophilic domains) that form an indispensable component in almost every sector of modern industry. Their significance is evidenced from the enormous volumes that are used and wide diversity of applications they are used in, ranging from food and beverage, agriculture, public health, healthcare/medicine, textiles, and bioremediation. A major drive in recent decades has been toward the discovery of surfactants from biological/natural sources—namely bio-surfactants—as most surfactants that are used today for industrial applications are synthetically-manufactured via organo-chemical synthesis using petrochemicals as precursors. This is problematic, not only because they are derived from non-renewable resources, but also because of their environmental incompatibility and potential toxicological effects to humans and other organisms. This is timely as one of today's key challenges is to reduce our reliance on fossil fuels (oil, coal, gas) and to move toward using renewable and sustainable sources. Considering the enormous genetic diversity that microorganisms possess, they offer considerable promise in producing novel types of biosurfactants for replacing those that are produced from organo-chemical synthesis, and the marine environment offers enormous potential in this respect. In this review, we begin with an overview of the different types of microbial-produced biosurfactants and their applications. The remainder of this review discusses the current state of knowledge and trends in the usage of biosurfactants by the Oil and Gas industry for enhancing oil recovery from exhausted oil fields and as dispersants for combatting oil spills.

## Introduction

Over the past 10 years, there has been a marked surge of interest and directed research activities toward the development of biosurfactants for applications in the Oil and Gas industry, so this review is largely dedicated to providing an update and current state-of-the-art on this emerging field. To begin with, there are two important reasons for this surge in interest, one of which stems from the fact that the oil production rate, which also accounts for the rate of discovering new underground oil reserves, has been in a steady state of decline for some decades now. The total quantity of underground oil reserves around the world is finite, so the rate of its production must eventually reach a peak (*aka* the Hubbard peak), which it already has. Thereafter over time, this rate has followed a decreasing trend, which has further spurred increased interests into the application of biosurfactants for the recovery of residual crude oil by a process called microbial enhanced oil recovery using microorganisms or their products (MEOR). The other main reason fuelling this surge in interest into developing biosurfactants for the Oil and Gas industry relates to debate regarding the use of synthetic chemical surfactants that are found as ingredients in chemical dispersant formulations. Chemical dispersants are commonly used by the industry as a first-step response tool for treating oil spills at sea. The dispersants that are approved and stockpiled worldwide for use in the event of an oil spill are manufactured by organo-chemical synthesis. Historically, the use of chemical dispersants has not always been documented or accurately recorded during oil spills. However, records exist for some high-profile cases, such as the Torrey Canyon oil spill in 1967 where ~13,500 tons of a dispersant agent was used to clean up the oil (Law, 2011), and also the Sea Empress oil spill in 1996 where ~12 tons of the dispersant Corexit 9500 was applied (Lessard and DeMarco, 2000). Dispersants were applied in other small-scale marine oil spills but their effectiveness in some occasions has been disputed, such as in the case of the Exxon Valdez spill in 1989 where the dispersant Corexit was applied inefficiently and later observed to be ineffective in cold environments. The dispersant toxicity to marine life has come a long way since the first studies back in the 1960s (Portmann and Connor, 1968), but their use does not fail to raise controversy and debate, and even more so following the Deepwater Horizon oil spill which was the first oil spill where dispersants were applied in significant quantities. Following the onset of this spill, at the direction of the Federal On-Scene Coordinator, unprecedented quantities, up to 7 million liters, of the dispersant Corexit EC9500A were applied by spraying on sea surface oil slicks and subsequently directly injected at the leaky wellhead near the seafloor (National Commission on the BP Deepwater Horizon Oil Spill Offshore Drilling, 2011); this was after the dispersant Corexit 9527 was used initially. This subsurface injection of Corexit resulted in droplet size distributions of ~10–30 μm in diameter in the deep-water oil plume, which significantly facilitated biodegradation (Brakstad et al., 2015). However, some studies showed that the use of Corexit inhibited the enrichment of some oil-degrading bacteria (Hamdan and Fulmer, 2011; Kleindienst et al., 2015; Rahsepar et al., 2016). Other studies showed no such inhibition or negative impacts from chemical dispersants (McFarlin et al., 2014; Brakstad et al., 2018). Whilst more research is needed to better understand the effects of synthetic chemical dispersants, such as Corexit, on the microbial response and the overall biodegradation of the oil, the Deepwater Horizon spill fuelled an unprecedented level of interest over these past 10 years since its onset to searching for alternative types of dispersants that are based on biosurfactants and which boast a greater environmental compatibility.

There are many reviews on the topic of biosurfactants, so we want to be upfront from the start and state that our intention here is not to confound the literature with yet, again, just another review. In this review, we begin with an overview on some of the most important biosurfactants that have been discovered to-date along with their commercial uses in order to set the scene on what is available and usable with respect to these incredibly versatile biomolecules. The remainder of this review is dedicated to presenting the current knowledge and state-of-the-art on these biomolecules for applications in MEOR, and also as dispersant agents for combatting oil spills at sea—two areas which have increasingly been given attention in recent years.

## Types of Biosurfactants

There are countless reviews that provide a detailed description of what defines a biosurfactant, or more generally “surface-active agents” which also encompasses emulsifiers (biomulsifiers from biological sources). Hence, we will not go into any length of detail here but offer the reader a good review on the topic by Desai and Banat (1997). Briefly, biosurfactants are surface-active, amphiphilic compounds derived from biological sources (microorganisms, plants, or animals). Generally, microorganisms such as bacteria, yeast, and archaea are the most commercially promising and sustainable source of surface-active compounds owing to their enormous genomic diversity. These compounds can form part of microorganism's cell wall, or are excreted extracellularly out of the cell (Shahaliyan et al., 2015). Because of their amphiphilic nature, biosurfactants can dissolve in both polar and non-polar solvents (Chen et al., 2015). The effectiveness of a surfactant is determined by its ability to lower the surface tension (ST) and interfacial tension (IFT) between two immiscible phases (e.g., air/liquid and non-polar/polar liquids, respectively). The ST is a measure of the energy (per unit area) required to increase the surface area of a liquid due to intermolecular forces. When a surfactant is present, less work is required to bring a molecule to the surface and consequently the ST is reduced. It is accepted that a good biosurfactant can lower the ST of water from 72 to <35 mN/m, and the IFT for water against *n*-hexadecane from 40 to 1 mN/m (Mulligan, 2005). An efficient biosurfactant is one that has a low critical micelle concentration (CMC). By definition, the CMC is the minimum concentration required to initiate micelle formation and generally correlates with the ST and IFT (Mulligan, 2009); in other words, a low CMC means that less biosurfactant is necessary to reduce the ST or IFT.

The chemical composition of biosurfactants varies greatly between different species of microorganisms and broadly can be classified based on their molecular weight or chemical charge. Based on their molecular weight, surface-active compounds are classified as either low-molecular-weight (LMW) surfactants, which reduce surface tension between two immiscible liquids, or high-molecular-weight (HMW) emulsifiers, which enable the formation of oil-in-water or water-in-oil emulsions and which are also referred to as polymeric surfactants (or bioemulsifiers) and commonly composed of exopolysaccharides (EPS).

The main chemical structures of LMW biosurfactants are glycolipids, phospholipids and fatty acids, lipopeptide, and lipoproteins. These structures can form biosurfactant as single macromolecules, polymers, and/or particulate structures (Banat, 1995; Makkar and Rockne, 2003; Satpute et al., 2010). Being of LMW, their main function as surface-active agents is in lowering of surface and/or interfacial tension between two immiscible phases of liquid, liquid and solid, or liquid and gas. Glycolipids are the most studied biosurfactants and consist of different sugars linked to ß-hydroxy fatty acids (carbohydrate head and a lipid tail), while lipopeptides consist of cycloheptapeptides with amino acids linked to fatty acids of different chain lengths (Table 1) (Uzoigwe et al., 2015).

**Table 1:** Summary of biosurfactant types based on their molecular weight, origin and industrial application. Wherever possible a schematic of the chemical structure is provided.

**Biosurfactant type**	**Origin**	**Chemical structure**	**Industrial application**	**References**
**Low-molecular weight biosurfactants**				
**Glycolipids**				
Rhamnolipid	*Pseudomonas aeruginosa* *Burkholderia thailandensis* *Marinobacter sp*. strain MCTG107b	Rhamnose monosaccharide/s linked to 3-hydroxyl fatty acid unit via ß-glycosidic bond	Bioremediation Agriculture Cosmetics Pharmaceuticals **Marine oil spills**	Funston et al., 2016; Chong and Li, 2017; Twigg et al., 2018; Tripathi et al., 2019
		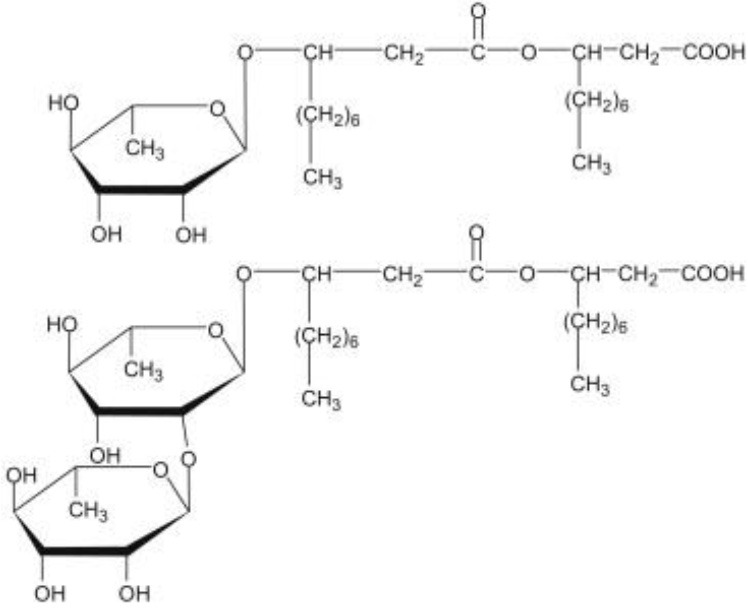		
Sophorolipid	*Candida* spp.	Dimeric sugar sophorose head linked to a long chain hydroxy fatty acid tail	Cosmetics Personal care products	Van Bogaert et al., 2007; Kurtzman et al., 2010; Santos et al., 2017
		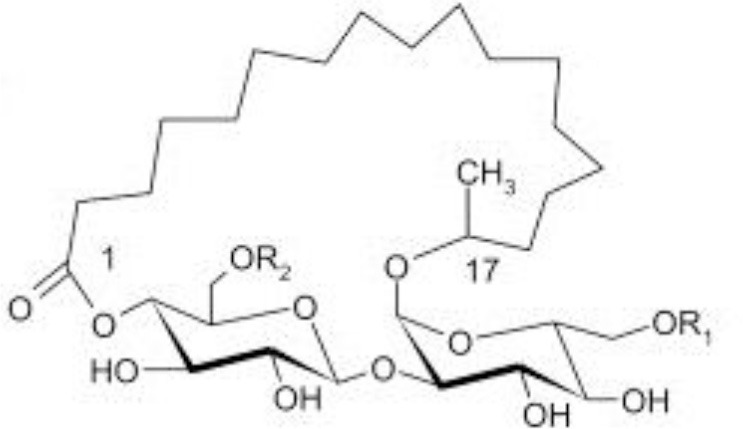		
Trehalose lipids	*Rhodococcus erythropolis*	Trehalose sugar linked to long chain fatty acids (C_20_ to C_90_)		Peng et al., 2007
		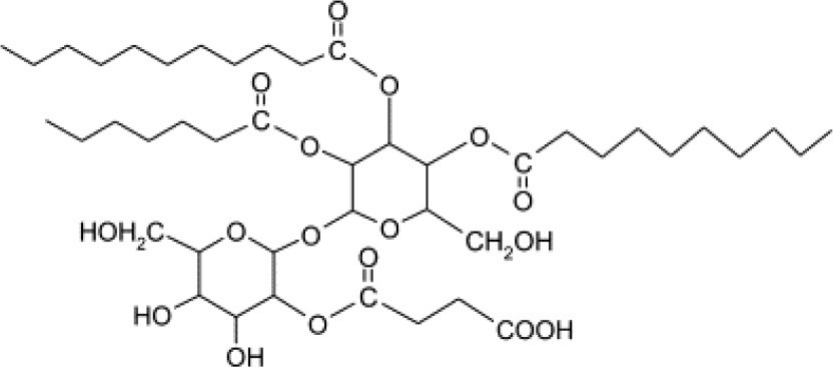		
Mannosylerythrol lipids (MELs)	*Candida antarctica* *Pseudozyma* spp.	Long-chain fatty acids linked to a mannopyranosyl-meso-erythritol hydrophilic head group	Food	Adamczak and Bednarski, 2000; Morita et al., 2013; Niu et al., 2019
		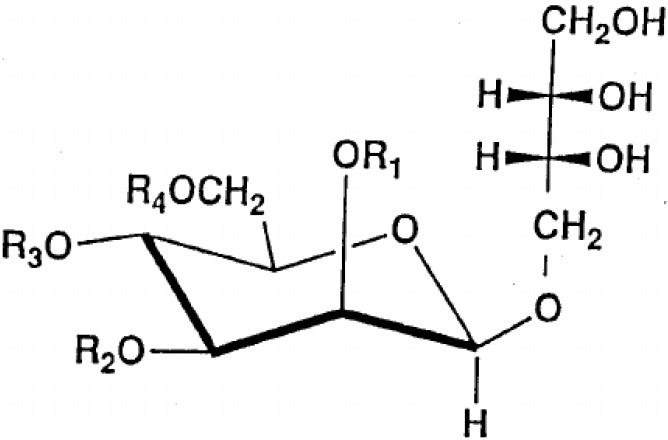		
**Lipopeptides**				
Surfactin, iturin and fengycin	*Bacillus subtilis*	Cyclic lipopeptide consisting of long hydroxyl fatty acid chain and hydrophobic amino acid ring	Soil bioremediation **MEOR**	Vanittanakom et al., 1986; Al-Wahaibi et al., 2014; Inès and Dhouha, 2015; Liu et al., 2015
		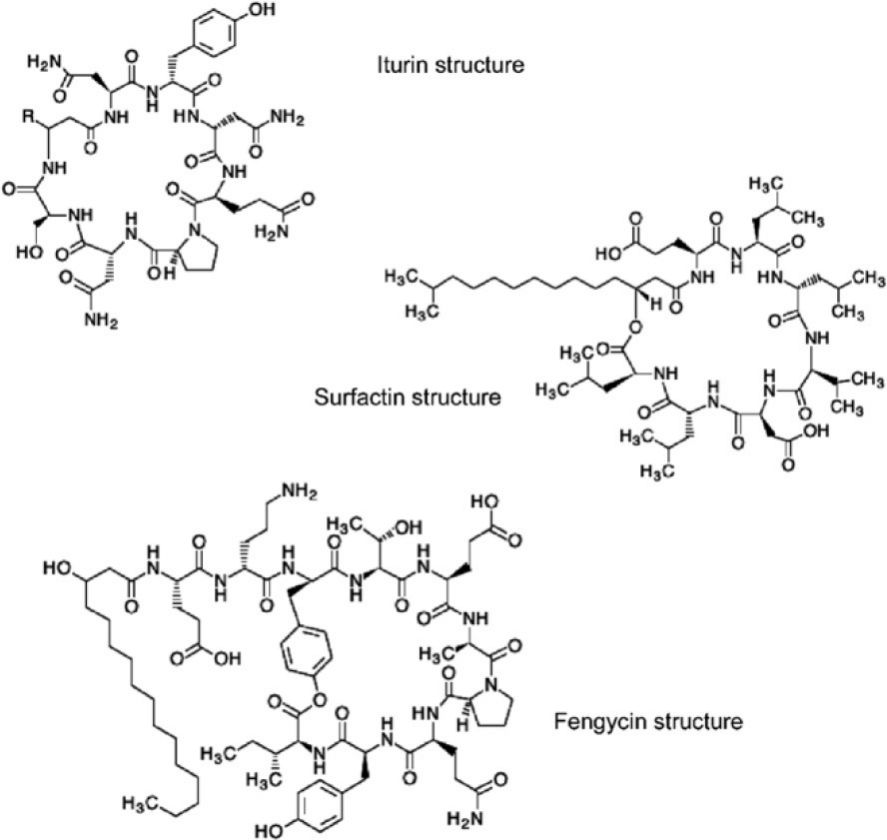		
Lichenysin	*Bacillus lichenformis*	Cyclic lipopeptide similar to surfactin	**MEOR**	Yakimov et al., 1995; Joshi et al., 2015; Coronel-León et al., 2016
Viscosin	*Pseudomonas fluorescens*	Cyclic lipopeptide similar to surfactin	Agriculture	Laycock et al., 1991; De Bruijn and Raaijmakers, 2009
**High-molecular weight bioemulsifiers**				
Emulsan	*Acinetobacter calcoaceticus* RAG-1	A complex of a lipoheteropolysaccharide (apoemulsan) and a protein	**MEOR**	Rosenberg and Ron, 1999; Uzoigwe et al., 2015
		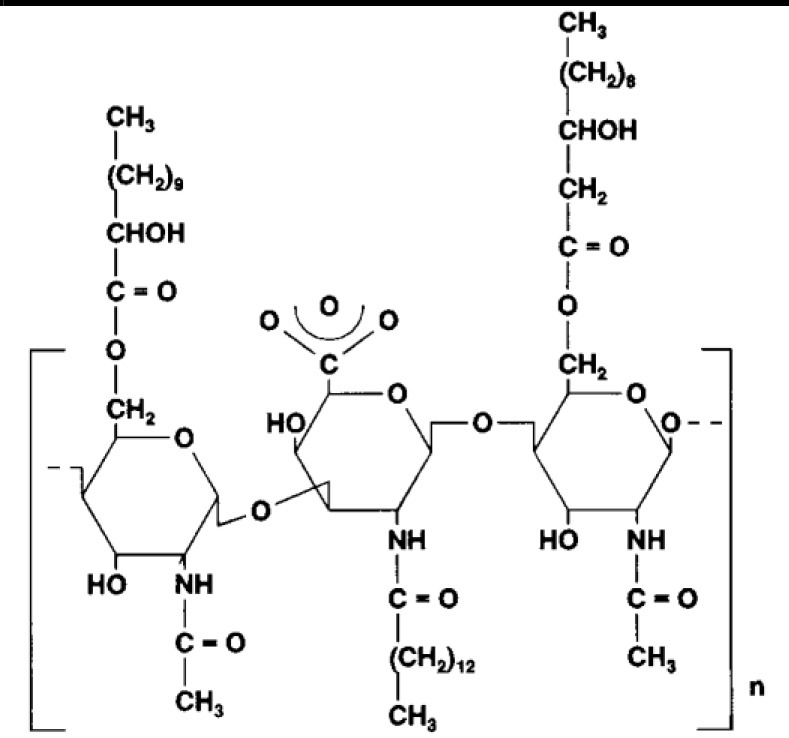		
Alasan	*Acinetobacter radioresistens* KA53	A complex of anionic polysaccharides rich in alanine and proteins with high molecular mass	Bioremediation **MEOR**	Navon-Venezia et al., 1995; Toren et al., 2001
Liposan	*Candida lipolytica*	A complex of heterpolysaccharides and protein	Pharmaceuticals Food Cosmetics	Cirigliano and Carman, 1985; Campos et al., 2013
Sphingan	*Sphingomonas spp*.	Linear tetrasaccharide backbone (glucose-glucoronic acid-glucose-rhamnose/mannose) to which glucosyl, rhamnosyl, mannosyl or acetyl side chains are attached	Food Textile Pharmaceuticals Oil and Gas	Schultheis et al., 2008; Prajapati et al., 2013; Kaur et al., 2014; Li et al., 2016
Xanthan gum	*Xanthomonas campestris*	A backbone of repeating sub-units of 3 to 8 monosaccharides	Food Oil and Gas	Kuppuswami, 2014; de Mello Luvielmo et al., 2016; Kang et al., 2019
		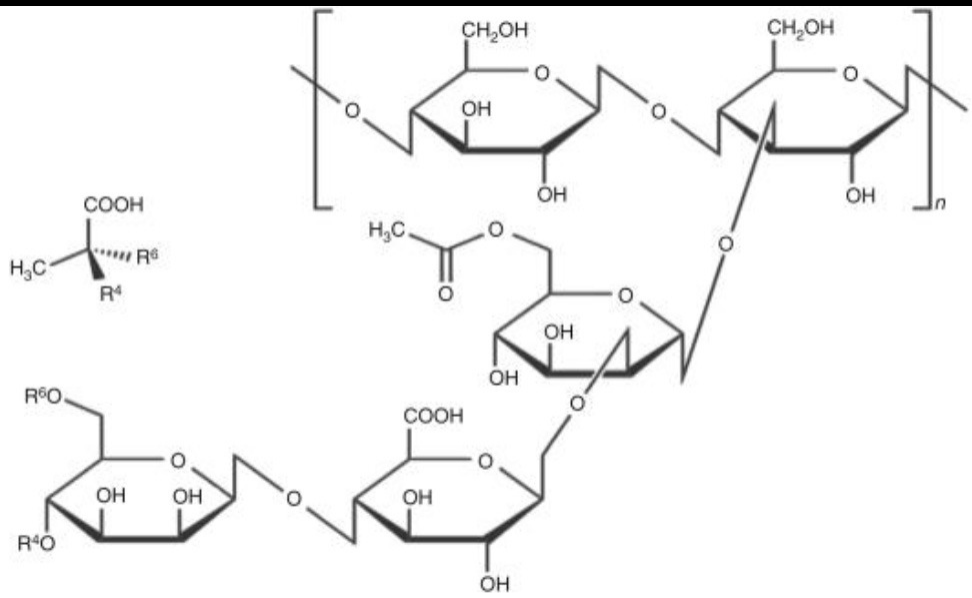		

High-molecular weight bioemulsifiers are more complex than biosurfactants and consist of mixtures of heteropolysaccharides, lipopolysaccharides, lipoproteins, and proteins. They are also known as EPS. Similar to low-molecular weight biosurfactants, EPS molecules can efficiently emulsify two immiscible liquids (e.g., oil and water), but in contrast are less effective at ST reduction. In oil polluted environments, EPS molecules bind tightly to dispersed hydrocarbons preventing the oil droplets from coalescing and “bursting” open. This process is known as stabilization of emulsion and has been attributed to the large number of reactive groups exposed in their structures (Uzoigwe et al., 2015). The best-studied microbial EPS are emulsan, alasan, liposan, sphingan, and xanthan gum (Table 1).

## Marine Biosurfactant-Producing Bacteria

A somewhat constrained diversity of bacterial species has been isolated from marine environments with the ability to produce biosurfactants that have found a commercial use or identified with potential in this respect. Whilst some species were originally isolated from terrestrial environments, as in the case of *Pseudomonas* and *Rhodococcus*, members of these genera with biosurfactant-producing qualities have subsequently been isolated from marine environments. These organisms and the properties of their produced biosurfactants are described below and summarized together with other lesser known marine biosurfactant producers in Table 2.

**Table 2:** Biosurfactant-producing bacteria from marine environments, hydrocarbon-polluted soil, oil reservoirs, or other origin.

**Environment**	**Organism**	**Carbon source**	**ST** **(mN/m)**	**EI_**24**_** **(%)**	**Chemical composition of surfactant**	**References**
Marine	*Alcanivorax borkumensis*	Alkanes	29		Glucose-lipid	Yakimov et al., 1998
Marine	*Alcanivorax dieselolei*	Alkanes	35	51	Glycolipid	Hassanshahian et al., 2012
Marine hydrothermal vent	*Alteromonas infernus*	Glucose			Acidic EPS	Raguénès et al., 1997b
Marine hydrothermal vent	*Alteromonas macleodii*	Glucose			Sulphated EPS	Raguénès et al., 1996; Rougeaux et al., 1998
Marine	*Arthrobacter sp*. EK1	Alkanes			Trehalose lipid	Passeri et al., 1991
Oiled soil	*Bacillus cereus* IAF 346	Sucrose	28	60	EPS	Cooper and Goldenberg, 1987
Oil reservoir	*Bacillus methylotrophicus* USTBa	Alkanes, PAH	28		Glycolipid	Chandankere et al., 2014
Oiled soil	*Bacillus pumilus* 2IR	Crude oil/glucose	32	>60	Lipopeptide	Fooladi et al., 2016
Oiled sludge	*Brevibacillus sp*. PDM-3	Phenanthrene	47	57	Glycolipid	Reddy et al., 2010
Other	*Burkholderia thailandensis*	Glycerol	32	42	Rhamnolipid	Díaz De Rienzo et al., 2016
Oil reservoir	*Clostridium sp*. N-4	Sucrose	32		Glycoprotein	Arora et al., 2019
Marine	*Cobetia sp*. MM1IDA2H-1	PAH	33	44	Lipidic surfactant	Ibacache-Quiroga et al., 2013
Marine	*Colwellia psychrerythraea* 34H				Capsular EPS	Casillo et al., 2017
Marine	*Corynebacterium kutscheri*	Motor oil; Peanut oil			Glycolipopeptide	Thavasi et al., 2007
Oil field	*Corynebacterium lepus*	Kerosene	30		Lipopeptide	Cooper et al., 1979
Marine	*Enterobacter cloaceae* 71a			>60	EPS	Iyer et al., 2006
Oiled soil	*Escherichia fergusonii* KLU01	Diesel oil	32		Lipopeptide	Sriram et al., 2011
Marine	*Flexibacter sp*. TG382	Glucose	67	>60	Glycoprotein EPS	Gutiérrez et al., 2009
Marine	*Gordonia amicalis* LH3	Paraffins, crude oil			Rhamnolipid	Hao et al., 2008
Marine	*Halomonas eurihalina*	Glucose, PAH		78	Sulfated heteropolysaccharide	Calvo et al., 2002
Marine	*Idiomarina sp*. 185	Tetradecane	29	30	Glycolipid	Malavenda et al., 2015
Marine	*Pantoea sp*. A-13	Paraffins, kerosene	30		Glycolipid	Vasileva-Tonkova and Gesheva, 2007
Marine sediment	*Pseudoaltermonas sp*. 93	Tetradecane	63	55	Glycolipid EPS	Malavenda et al., 2015
Oil reservoir	*Pseudomonas aeruginosa* WJ-1	Alkanes, Veg oils	24	100	Rhamnolipid	Xia et al., 2013
Other	*Pseudomonas cepacian* CCT6659	Veg oil	27–29	90–100	Rhamnolipid	Soares da Silva et al., 2019
Marine hydrothermal vent	*Vibrio diabolicus* HE800	Glucose			EPS	Raguénès et al., 1997a
Oiled soil; marine	*Virgibacillus salanis*	Glucose	30	80	Lipopeptide	Elazzazy et al., 2015

*Empty fields mean that these parameters were not determined by the source*.

### Pseudomonas

Members of the genus *Pseudomonas*, of the class Gammaproteobacteria, can colonize diverse habitats and produce different biosurfactant molecules including glycolipids (rhamnolipids) and lipopeptides (e.g., viscosin, amphisin, tolaasin, and syringomycin). The majority of isolated *Pseudomonas* species have been derived from terrestrial habitats, but members of this genus are common in marine environments (Baumann et al., 1983; Bollinger et al., 2020). The most commonly studied biosurfactant producer is *P. aeruginosa*. It grows well on various hydrocarbon and non-hydrocarbon substrates and produces rhamnolipids that can form stable emulsions with crude oil and kerosene (Shahaliyan et al., 2015). Another study observed that *P. aeruginosa* had a high affinity for crude oil (93% cell adhesion to crude oil) which is also an indication of biosurfactant production (Thavasi et al., 2011). *P. aeruginosa* DQ8 strain was shown to decrease the surface tension from 63 to 38 mN/m of culture broth in the presence of various crude oil fractions including PAHs (Zhang et al., 2011). In addition, non-hydrocarbon substrates such as soybean oil, fish oil, mannitol, and glycerol, can be utilized by *P. aeruginosa* to produce non-toxic biosurfactant which could be useful in oil spill bioremediation as an alternative to chemical dispersants or as substitute of synthetic surfactants in commercial dispersant formulations (Coelho et al., 2003; Prieto et al., 2008; Das et al., 2014). Strains of *P. aeruginosa* grown on glycerol produced rhamnolipids (3.8 g/L; CMC 50 mg/L) which reduced the ST to 29 mN/m and emulsified petrol (EI_24_ 70%) and diesel (EI_24_ 80%), further indicating its potential application in oil recovery and bioremediation (Das et al., 2014). A species of *Pseudomonas putida*, stain BD2, was isolated from Arctic soil that was able to grow on glucose and produce rhamnolipid and sophorolipid simultaneously; the rhamnolipid reduced ST to 31 mN/m and emulsified veg oil at 70% efficiency (Janek et al., 2013).

### Bacillus

Members of the genus *Bacillus* have been predominantly isolated from oil reservoirs or oil contaminated soils and shown to be particularly efficient biosurfactant producers with applications in MEOR. *Bacillus methylotrophicus* USTBa, for example, was isolated from a petroleum reservoir and grew well on crude oil in aqueous medium. After 12 days of incubation, *B. methylotrophicus* removed more than 90% of the crude oil. The ST of the culture medium was measured at 28 mN/m indicating that the bacteria produced a strong glycolipid-type biosurfactant (Chandankere et al., 2014). In addition, the biosurfactant was stable under various pH values and high temperatures (up to 100°C) suggesting its potential application as an oil spill treatment agent in marine environments and in MEOR processes where high salinity and temperatures are common (Chandankere et al., 2014). *B. subtilis* strain A1 was able to achieve 78% emulsification activity by the production of lipopeptide biosurfactant when grown on crude oil as a sole source of carbon (Parthipan et al., 2017). This strain completely degraded a range of the low-molecular weight alkanes (C_10_-C_14_) and up to 97% of the high-molecular weight alkanes (C_15_-C_19_) after 7 days of incubation at 40°C. These results suggest that *B. subtilis* A1 strain could be used in oil spill remediation where light crude oils (high proportion of alkanes) have been spilled. A non-pathogenic *B. licheniformis* R2 was studied for its potential use in MEOR in laboratory conditions. It produced a low-yield lipopeptide biosurfactant (1 g/L) that lowered the ST to 28 mN/m and the IFT between heavy crude oil and formation water-brine used in core flooding to 0.53 mN/m (Joshi et al., 2015). When treated with temperature of 85°C, the R2 biosurfactant recovered 37% heavy crude oil recovery over residual oil saturation and retained 88% activity for 90 days (Joshi et al., 2015).

### Acinetobacter

*Acinetobacter* is a genus of gram-negative Gammaproteobacteria, strictly aerobic bacteria belonging to the order of Pseudomonadales. *Acinetobacter* is ubiquitous in nature and commonly found in marine environments. Many species from this genus are known hydrocarbon-degraders that produce extracellular EPS (Pines and Gutnick, 1986; Barkay et al., 1999; Hassanshahian et al., 2012). As mentioned earlier, *A. calcoaceticus* and *A. radioresitensis* synthesize emulsan and alasan, respectively, well-known HMW bioemulsifiers (Kaplan et al., 1985; Navon-Venezia et al., 1995). *A. calcoaceticus* was also able to synthesize rhamnolipids with CMC of 15 mg/L (Hošková et al., 2015). *A. radioresitensis* can produce a yield of 4.6 g/L of EPS when grown on ethanol as the sole carbon and energy source (Navon-Venezia et al., 1995). Strains of *A. calcoaceticus*, and *A. oleivorans* isolated from the Canadian North Atlantic have been shown to produce bioemulsifiers when grown on petroleum hydrocarbons as the sole carbon source (E_24%_ > 50%) (Cai et al., 2014). Given that the strains were isolated and hence adapted to the cold marine environment of the Northern Atlantic, the bioemulsifiers they produce could be effective under low temperature and harsh conditions in offshore oil spills remediation.

### Antarctobacter

*Antarctobacter* is a genus of gram-negative bacteria (order Rhodobacterales), that are strictly aerobic bacteria. Only one species, *Antarctobacter heliothermus*, has been validly taxonomically described which was isolated from Antarctica (Labrenz et al., 1998). *Antarctobacter* sp. strain TG22 was isolated from seawater and was found to produce an extracellular water-soluble glycoprotein-type polymer (designated AE22) which formed stable emulsions with different vegetable oils at concentration as low as 0.02% (Gutiérrez et al., 2007a). The strain was grown on marine broth supplemented with 1% glucose and was able to produce an average dry-weight yield of 21 mg/L. The carbohydrate content (total of 15%) of AE22 was dominated by glucosamine, glucuronic acid, fucose and mannose. The protein content represented 5% of the polymer and lipids were not detected, leaving the rest of the polymer content (80%) unidentified (Gutiérrez et al., 2007a). The emulsifying activity of *Antarctobacter* TG22 polymer was comparable to that of xanthan gum which could be considerably useful in applications for healthcare and food additives industries.

### Rhodococcus

The genus *Rhodococcus* includes metabolically diverse species that are capable to thrive in different habitats (Finnerty, 1992). Members of the genus have been studied mainly for their ability to degrade hydrocarbons and pollutants from different environments (Whyte et al., 2002; Kuhn et al., 2009; Wang et al., 2014). *Rhodococcus erythropolis, Rhodococcus aurantiacus*, and *Rhodococcus ruber* are among the best known biosurfactant producers of the genus (Bicca et al., 1999; Peng et al., 2007). *R. erythropolis* 3C-9 has been shown to grow and produce biosurfactant (CMC of 50 mg/L) only on *n*-alkanes as the sole carbon source, whereas glucose could not enhance its productivity. The 3C-9 biosurfactant contained fatty acids with lengths from C_10_ to C_22_ (docosenoic acid being the most prevalent followed by hexadecenoic acid) and two glycolipids (each dominated by glucose and trehalose monosaccharides). In addition, the 3C-9 biosurfactant significantly enhanced the solubility of PAH substrates (Peng et al., 2007). *R. ruber* stain AC 239 produced a small amount of cell-bound glycolipid-type biosurfactant when grown on 1% diesel (v/v). The AC 239 biosurfactant did not reduce the ST as observed for other glycolipid biosurfactants but it emulsified different hydrocarbons with better success (20–50% greater EI_24_) when free cells were present in the culture (Bicca et al., 1999). *R. fascians* extracted from Antarctic soil produced a glycolipid with rhamnose sugars which is not typical for *Rhodococcus* which usually produces trehalose biosurfactants (Gesheva et al., 2010).

### Halomonas

*Halomonas* is a ubiquitous genus of the order Altreromonadales. There organisms are found in diverse habitats of both marine (Hassanshahian et al., 2012; Cai et al., 2014) and terrestrial environments, including hypersaline lakes (Poli et al., 2004), soils (Arias et al., 2003; Mata et al., 2006; Llamas et al., 2012; Amjres et al., 2015), and hot springs (Chikkanna et al., 2018). Members of *Halomonas* are known to respond to hydrocarbon enrichment (Calvo et al., 2002; Gutierrez et al., 2013; Cai et al., 2014) and produce EPS (Gutiérrez et al., 2007b; Gutierrez et al., 2009, 2020) with versatile properties. A thermophilic *H. nitroreducens* strain WB1 isolated from a hot spring produced an EPS that was effective at emulsifying different vegetable oils (68–85%) and aliphatic hydrocarbons (56–65%) in addition to binding metals. The monosaccharide composition of the WB1's EPS was predominantly composed of glucose, mannose and galactose, and traces of uronic acids (Chikkanna et al., 2018). The EPS from *Halomonas eurihalina* strain H96, isolated from saline soil in Spain, has been characterized to contain high amount of uronic acids (Béjar et al., 1998) similarly to some marine-derived strains (Gutierrez et al., 2020). In addition to emulsifying activity, several species of halophilic *Halomonas* have been shown to produce highly sulphated exopolysaccharides (Calvo et al., 2002; Amjres et al., 2015) with anticancer activity. For example, halophilic *H. stenophila* strain B100 exerted a selective proapoptotic effect in T cells from acute lymphoblastic leukemia (Ruiz-Ruiz et al., 2011). EPS form *H. halocynthiae* KMM 1376 had inhibitory effect on human cancer cell line MDA-MB-231 at concentrations of 50–100 μg/ml (Kokoulin et al., 2020).

### Alcanivorax

*Alcanirorax* is gram-negative genus of the Gammaproteobacteria (order Oceanospirillales) of strictly aerobic marine obligate hydrocarbonoclasic bacteria (OHCB) utilizing predominantly alkanes up to C_32_ and branched aliphatics (Head et al., 2006; Yakimov et al., 2007; Olivera et al., 2009). The best known species of the genera is *Alcanivorax borkumensis* which produces a low molecular weight anionic glycolipid biosurfactant when grown on hydrocarbons (Schneiker et al., 2006). This particular glycolipid consists a glucose sugar linked to a tetrameric chain of fatty acids of C_6_-C_10_ length and can be either cell-bound or extracellular (Abraham et al., 1998). Marine isolate *A. borkumensis* SK2 grown on crude oil produced twice more biosurfactant than in the absence of hydrocarbons. In fact, when heavy hydrocarbon fractions were used as the sole carbon source, biosurfactant was the highest (~70 mg/L) and comparable with when crude oil was used (50 ± 20 mg/L). However, the purification of the biosurfactant was easier when the culture was fed with heavy oil fraction as it remained on the surface at all times and consequently there were no substrate impurities (Antoniou et al., 2015). Another species, *A. dieselolei* strain B-5, is the second in the genus that has been reported to produce biosurfactant with good surface-active properties (lower ST to 32 mN/m and emulsify *n*-hexadecane at 75%). The chemical analysis of the biosurfactant revealed that it is a linear lipopeptide with CMC value of 40 mg/L which is comparable to that of rhamnolipids and surfactin (Qiao and Shao, 2010). These characteristics make the B-5 lipopeptide an attractive alternative for enhanced oil recovery and bioremediation applications.

### Pseudoalteromonas

*Pseudoalteromonas* is a genus of the order Alteromonadales, members of which are commonly found in sea ice and cold waters and well-known producers of glycolipid-type EPS with a wide-range of biological activities and chemical composition (Holmström and Kjelleberg, 1999). *Pseudoalteromonas* sp. strain SM20310 isolated from Arctic sea ice produced EPS (yield of 567 mg/L) with mannose and glucose being the dominant carbohydrates. The ecological role of the EPS was determined to improve the high-salinity and low-temperature tolerance of the strain (Liu et al., 2013). Another study found that marine *Pseudoalteromonas* (isolated from Antarctica) produced EPS that contained 40% protein with mannose, glucose and galacturonic acid representing the dominant monosaccharides (Nichols et al., 2005). The carbohydrate content of EPS from *P. agarivorans* stain Hao 2018 (yield 4.5 g/L) isolated from Yellow Sea of China contained 90% glucose and 6% mannose. The main biological activity of this strain was moisture retention and absorption of free radicals (i.e., antioxidant) with potential applications in food and cosmetics industries (Hao et al., 2019). *Pseudoalteromonas* sp. strain MD12-642 (isolated from Madeira) produced EPS with a particularly high content of uronic acids (up to 68%) and which might find potential applications in biomedical industry as active ingredients for anti-thrombotic and anti-arthritic drugs (Roca et al., 2016). *Pseudoalteromonas* sp. strain TG12 (isolated from West Scotland) produced EPS that was able to effectively emulsify *n*-hexadecane (EI_24_ of 60%) and some vegetable oils. This strain also contained high levels of uronic acids (~29%) in addition to xylose (27%), glucosamine (25%) and was effective in desorption of sediment-adsorbed metals (e.g., Al^3+^, Fe^2+/3+^, K^+^, Mg^2+^, Na^+^, and Si^4+^) (Gutierrez et al., 2008).

### Marinobacter

*Marinobacter* is a genus within the order Alteromonadales. Members of the genus, such as *M. hydrocarbonoclasticus* and *M. algicola* are commonly isolated from oil-enriched marine environments (Gauthier et al., 1992; Gutierrez et al., 2013). Although *Marinobacter* can use hydrocarbons as carbon source, various studies demonstrated that it can also grow and produce EPS on other carbon sources such as glucose. *Marinobacter* species have been shown to produce exopolysaccharide polymers with excellent emulsifying activity against hydrocarbons that were superior to commercial synthetic surfactants like Tween 80 (Caruso et al., 2019). *Marinobacter* sp. W1-16 from Antarctic surface seawater produced EPS (molecular weight of 260 kDa) with varying yields, strongly depending on the sugar substrate used to grow the strain and the incubation temperature. The highest yield was produced when the culture was grown at 15°C and in the presence of 2% glucose. However, the strain was able to synthesize EPS, even at 4°C, albeit in lower quantities, suggesting that the EPS might have cryoprotective functions (Caruso et al., 2019). In addition, *Marinobacter* sp. MCTG107b was able to produce a glycolipid-type biosurfactant (grown on glucose) with di-rhamnolipid congeners present that was able to lower the ST to 30 mN/m (Tripathi et al., 2019). Marine sediment isolates belonging to *Marinobacter* genus produced (when grown on glucose or soybean oil) powerful emulsifiers with activity against hexane and toluene in the range of 45–64 and 33–75%, respectively, with some strains producing stable emulsions at 4°C and after high-temperature treatment for up to 18 months (Raddadi et al., 2017).

## Ecology and Environment

Biosurfactant-producing bacteria can be found in a wide range of habitats, from aquatic (fresh and sea water, and groundwater) to terrestrial (soil, sediment, and sludge) environments. Extreme environments, characterized by extremes of high/low temperature, salinities, pH, and/or pressure, are also commonplace where biosurfactant-producing microbes can be found due to the ecological role biosurfactants play for the producing organisms in those environments, such as in cell adhesion to surfaces and potential food sources, retention of water and concentration of nutrients, production of biofilms etc. (Nicolaus et al., 2010). The environment can have a direct influence on the type of biosurfactants that microorganisms produce. For instance, marine microbial EPS contain higher levels of uronic acids which makes them polyanionic and relatively highly reactive (Gutiérrez et al., 2007b; Decho and Gutierrez, 2017) compared to non-marine microbial surfactants. With respect to cold-adapted bacteria, the monosaccharides in the EPS are usually characterized by the presence of mannose and galactosamine (Nichols et al., 2004, 2005). Examples of extreme environments from which biosurfactant-producing microbes have been isolated and cultured under laboratory conditions include oil reservoirs (Arora et al., 2019; references in Nikolova and Gutierrez, 2020), cold environments (e.g., polar regions) (Gesheva et al., 2010; Malavenda et al., 2015; Casillo et al., 2018; Perfumo et al., 2018), salt lakes (Béjar et al., 1998; Amjres et al., 2015), and hydrothermal vents (Raguénès et al., 1996; Rougeaux et al., 1998). Moreover, the most obvious place to search for marine biosurfactant-producing microorganisms is in hydrocarbon-polluted areas since biosurfactants play an important part in the process of microbial biodegradation of hydrocarbons (Chandankere et al., 2014).

Most biosurfactant-producing microorganisms can survive and even thrive in a wide range of temperatures, pH and salinity and therefore exhibit a wide range of metabolic processes. The majority of isolated and cultured microorganisms are aerobic because they are relatively easy to be sampled and handled in laboratory conditions. However, the number of biosurfactant-producing microorganisms, largely comprising anaerobes, that are being discovered and successfully cultured in *ex-situ* conditions is steadily growing (VanFossen et al., 2008). Anaerobic microorganisms are also able to produce biosurfactants and have typically been found in oil reservoirs where anaerobic hydrocarbon biodegradation processes occur through methanogenesis (Head et al., 2003; Jones et al., 2008). *B. lichenformis*, for example, can grow and produce biosurfactants under both aerobic and anaerobic conditions (Javaheri et al., 1985; Al-Sayegh et al., 2015), however, the biomass and surfactant yields under such conditions can significantly lower compared to under conditions (Yakimov et al., 1995).

## Current Exploitation of Biosurfactants for Oil and Gas Industry Applications

Biosurfactants are becoming important biotechnology products for many industrial applications including in food, cosmetics and cleaning products, pharmaceuticals and medicine, and oil and gas. The global market revenues generated by biosurfactants exceeded USD 1.5 billion in 2019 and is projected to grow at over 5.5% CARG^1^ between 2020 and 2026 (Ahuja and Singh, 2020). Household detergents are the largest application market, followed by cosmetics and personal care, and the food industry (Singh et al., 2018). Key manufacturers of biosurfactants include Ecover, Jeneil Biotech, Evonik, and Biotensidon among others (Table 3). Europe has over half of the market share followed by the United States and Asia (Singh et al., 2018). The increasing global interest in biosurfactants is due to their low toxicity, biodegradability, low environmental footprint and impact (Desai and Banat, 1997).

**Table 3:** Biosurfactants and polymeric surface-active agents, the companies producing these biomolecules and their respective industrial applications (not confined to just the Oil & Gas industry).

**Company (website)**	**Location**	**Products**	**Industry application**
AGAE Technologies LLC (www.agaetech.com)	USA	Rhamnolipids	Pharmaceuticals, cosmetics, personal care, bioremediation, EOR
Jeneil Biotech (www.jeneilbiotech.com)	USA	Rhamnolipids	Cleaning products, EOR
Biotensidon (www.biotensidon.com)	Germany	Rhamnolipids	Agriculture (plant pest control), cosmetics, cleaning products,EOR
Frauhofer IGB (www.igb.fraunhofer.de)	Germany	Glycolipids, MELs	Cleansing products (shower gels, shampoos, washing-up liquids)
Saraya Co. Ltd (worldwide.saraya.com)	Japan	Sophorolipids	Cleaning products, cosmetics, hygiene products
Ecover (www.ecover.com)	Belgium	Sophorolipids	Cleaning products, cosmetics, bioremediation, pest control, pharmaceuticals
Groupe Soliance (www.soliance.com)	France	Sophorolipids	Cosmetics
MG Intobio Co. Ltd	South Korea	Sophorolipids	Beauty and personal care
Evonik (corporate.evonik.com)	Germany	Sophorolipids	Cleansing products
Lipotec S.A.U (www.lipotec.com)	Spain	Marine EPS	Cosmetics, personal care
Biopolymer International (www.biopolymer-international.com)	Belgium	Xanthan and gellan gums	Food, personal care, pharmaceuticals, oil drilling, animal feed

*Adapted from Randhawa and Rahman (2014)*.

### Soil Bioremediation

Hydrocarbon soil contamination, such as from drilling, leaking pipelines, storage tanks, transportation etc., is a widespread problem with long lasting environmental impacts. Being highly hydrophobic, particularly when adsorbed onto soil particles, hydrocarbons, and heavy metals are very resistant to removal. Typically, a variety of physical and chemical treatments, such as removal, incineration, soil washing, and solvent extraction have been used successfully in the past. However, such techniques are deeply damaging to the soil structure and the autochthonous biodiversity, as well as cost-prohibitive. As such, bioremediation is the preferred soil treatment due to its efficiency, lower environmental impact, and cost-effectiveness. Bioremediation involves naturally occurring soil microorganisms which convert petroleum hydrocarbons into carbon dioxide, water, and cell biomass. There are many factors that influence the rate and extent of hydrocarbon degradation in soils, such as moisture content, aeration, pH, temperature, the biological condition of the soil (aged vs. fertile soils; nutrient content and bioavailability), and the concentration, molecular structure, and bioavailability of the hydrocarbon contaminants (Venosa and Zhu, 2003; Huesemann, 2004). The optimisation of these environmental factors is critical for the bioremediation success.

Soil bioremediation can be conducted either in place (i.e., *in-situ*), or the contaminated soil is upended and, transported to be subsequently treated elsewhere (*ex-situ*). *In-situ* bioremediation involves, generally, the treating of only the top 30-cm layer of the soil with fertilizers to stimulate indigenous soil microorganisms to break down the hydrocarbons (Atlas and Hazen, 2011). This treatment is the preferred method of choice, but the risk of contaminating underlying aquifers with dissolved hydrocarbons must be considered. Partially purified biosurfactants have been used *in-situ* to increase the solubility and bioavailability of hydrocarbons, and other hydrophobic contaminants, by increasing their surface area (Ron and Rosenberg, 2002; Bustamante et al., 2012). A field trial on LaTouche Island in Alaska demonstrated that a biologically derived surfactant, PES-51, could remove 30% of semi-volatile petroleum hydrocarbons from a subsurface beach material (Tumeo et al., 1994). However, the majority of bioremediation studies with biosurfactants are under laboratory conditions. A study from Argentina demonstrated that surfactin from *B. subtilis* strain O9 contributed to significantly more removal of crude oil from sandy loam soil than in soil without surfactin within a period of 300 days (Cubitto et al., 2004). The addition of rhamnolipid from *P. aeruginosa* strain SSC2 to crude oil-contaminated soil sludge resulted in 98% degradation after 4 weeks compared to the non-rhamnolipid control treatment (67%). The effect was enhanced by adding nutrients to the treatments (Cameotra and Singh, 2008). An *in-situ* experiment of soil bioremediation conducted near oil production facility in Pakistan demonstrated that higher crude oil degradation (up to 77%) was achieved in soil treated with a combination of a specialized bacterial consortium, rhamnolipids and nutrients (Tahseen et al., 2016). The efficiency of MELs produced by *Candida antarctica* SY16 to degrade crude oil in soil was investigated by Baek et al. (2007). The authors compared different bioremediation techniques (i.e., natural attenuation, biostimulation, bioaugmentation, biosurfactant addition, and a combination of all) and concluded that the combined treatment of biostimulation, bioaugmentation with oil degrading *Nocardia* sp. H17-1 and with MELs caused the highest total petroleum hydrocarbon degradation rate during the first 4 weeks of treatment. However, at the end of the experiment (100 days) the amount of residual hydrocarbons was similar for all treatments (Baek et al., 2007).

### Microbial Enhanced Oil Recovery (MEOR)

MEOR is a process in which microorganism and/or their metabolic by-products are injected into mature oil reservoirs for the recovery of residual crude oil that was not extracted during the initial and secondary extraction processes. The idea behind MEOR is that when favorable conditions are present in the reservoir, the introduced microbes grow exponentially and their metabolic products would mobilize the residual oil (Gao and Zekri, 2011). MEOR bares with it its advantages and limitations, and the various processes of its application have been described extensively in the literature and recently summarized by Nikolova and Gutierrez (2020).

MEOR is based on two fundamental principles. Firstly, oil movement through porous media (rock formation) is facilitated by altering the interfacial properties of the oil-water-minerals displacement efficiency (i.e., decrease in IFT to increase the permeability of media), driving force (reservoir pressure), fluidity (miscible flooding; viscosity reduction), and sweep efficiency (selective plugging; mobility control). The second principle constitutes the degradation but also the removal of sulfur and heavy metals from heavy oils, by microbial activity (Shibulal et al., 2014). In the majority of MEOR field trials, injection of indigenous (or other MEOR suitable) pre-cultured bacteria or a consortium of bacteria along with nutrients (e.g., oxygen, nitrogen) has been the preferred choice of method because bacteria can produce biosurfactant *in-situ* which in turn significantly reduces the operational costs (Lazar et al., 2007; Geetha et al., 2018). However, biosurfactants produced *ex-situ* can also be used to enhance the microbial growth in oil reservoirs. In addition, when poor oil recovery from an oil well is due to low permeability of the rock formation, or to the high viscosity of the crude oil, the ability of biosurfactants to reduce IFT between the flowing aqueous phase and the residual oil saturation can improve the recovery process (Brown, 2010). Reduction of IFT by biosurfactants can also reduce the capillary forces that prevent the oil from moving through rock pores, however, the decrease in IFT must be at least two orders of magnitude to achieve mobilization of the oil. Typically, IFT between hydrocarbons and water is between 30 and 40 mN/m. For biosurfactants to have any effect in MEOR, they must reduce the IFT to 10^−3^ mN/m (Gray et al., 2008). To our knowledge such values have not yet been reported for known biosurfactants, and hence the effectiveness of IFT reduction may be limited in practice. Moreover, considering the type of oil reservoir (sandstone, carbonates etc.), residual oil saturation and the incremental oil recovery, the volume of biosurfactant that needs to be injected to achieve 30–60% oil recovery rate could be quite substantial and hence not practical or economical (Kowalewski et al., 2006; Gray et al., 2008; Afrapoli et al., 2011). In addition to reduction of IFT, biosurfactants can alter the wettability of rock formations, emulsify the crude oil, and contribute to the microbial metabolism of viscous oil (Sen, 2010).

Nevertheless, there are some promising results reported from different research groups that investigate biosurfactants for MEOR applications. Of all known biosurfactants, lipopeptides were mostly used in laboratory-based MEOR studies due to their ability to reduce the IFT to below 0.1 mN/m (Youssef et al., 2007; Gudiña et al., 2012; Pereira et al., 2013). Both bench-scale and *in-situ* lipopeptide production by stains of *Bacillus* spp. have proven successful in improving the oil recovery, including from wells close to their production limits (Al-Wahaibi et al., 2014; Al-Sayegh et al., 2015). Surfactins have been shown to maintain activities under a wide range of temperature, pH, and salinity while able to recover sand trapped oil. For example, *B. subtilis* produced surfactin at high temperature which could emulsify diesel with 90% efficiency and recover over 60% of oil entrapped in sand core (Makkar and Cameotra, 1997). Surfactin was recently shown to alter the wettability of CO_2_ injected in a subsurface rock formation demonstrating its potential suitability in carbon capture and storage application (Park et al., 2017). Lichenysin was reported to reduce the IFT to values of <10^−2^ mN/m (even at low concentrations of 10–60 mg/L) and to have exceptional stability under temperatures as high as 140°C, a pH range from 6 to 10, at salinities up to 10% NaCl, and at calcium (as CaCl_2_) concentrations up to 340 mg/L. In core flooding experiments, partially purified lichenysin recovered up to 40% of residual oil from sandstone cores compared to 10% recovery when chemical surfactants were applied (McInerney et al., 1990). Addition of biosurfactants during chemical surfactant flooding can improve the flooding performance in general. In the presence of rhamnolipids, the adsorption to sandstone of the surfactant alkylbenzene sulfonate was reduced by 25–30% and the quality of oil recovery increased by 7%. It has been suggested that rhamnolipids act as sacrificial agents by preferably adsorbing to the oil sands, making the surfactant more available for displacement activity and resulting in altering the wettability of porous media (Perfumo et al., 2010). Macromolecular biopolymers such as emulsan were shown to remove up to 98% of pre-adsorbed crude oil to limestone core samples, even at low concentration of 0.5 mg/ml (Gutnik et al., 1983). Recently, another biopolymer produced by *Rhizobium viscosum* CECT908 showed better efficiency than xanthan gum in the recovery of heavy oil (Couto et al., 2019).

### Marine Oil Spill Response

Crude oil is highly hydrophobic and it is composed of thousands of hydrocarbon and non-hydrocarbon species and metals, each with their respective aqueous solubilities. When an oil is introduced into a water phase, it will float on the surface of the water phase due to its lower density relative to water. Together with viscosity, surface tension is an indication of how rapidly and to what extent an oil spreads over the surface and, when dispersed, within the subsurface. The lower the interfacial tension with water, the greater is the extend of spreading (Fingas, 2011). To increase the solubility of oil in water (i.e., to decrease the surface tension between oil and water), chemicals are applied to an oil slick (Brakstad et al., 2015). Dispersed oil is usually in the form of fine neutrally buoyant droplets with higher surface area-to-volume ratio (diameter size 1–70 μm) compared to non-dispersed oil, thus making the oil available for biodegradation by hydrocarbon-degrading bacteria (ITOPF, 2011). The natural fate of crude oil biodegradation (biological oxidation by microorganisms) in the marine environment has been extensively described (Atlas and Hazen, 2011; Kostka et al., 2011; Campo et al., 2013; Prince et al., 2013; Wade et al., 2013; Hazen et al., 2016; Joye et al., 2016; Seidel et al., 2016).

Marine bioremediation research has largely been limited to application of fertilizers and/or seed cultures of highly efficient oil-degrading microorganisms, though with conflicting results (Prince, 2010). The limitation of marine oil remediation when relying solely on indigenous microorganisms is that the concentrations of cells in oil-polluted open water systems is never high enough to effectively emulsify oil (Ron and Rosenberg, 2002). The addition of surfactants (biogenic or synthetically produced) aims to disperse/emulsify the oil and, in turn, speed up the biodegradation process. Biosurfactants, have been shown to be effective in dispersing crude oil and enhancing the biodegradation process only under laboratory conditions due to logistical, financial and regulatory limitations of conducting large-scale field trials. In a laboratory study, a bacterial consortium containing oil degrading strains of *Ochrobactrum* and *Brevibacillus* with/without rhamnolipids were added to crude oil in 1 L water tanks to stimulate marine oil spill bioremediation. The results showed that the removal efficiency of oil by the bacterial consortium alone was lower by 6% compared to the combination of consortium and biosurfactant (Chen et al., 2013). The authors also noted that the presence of rhamnolipid enhanced the biodegradation of alkanes of chain length > *n*C_15_, but, interestingly, had the opposite effect on shorter chain alkanes (*n*C_13_-C_15_) by reducing their solubility and increasing their stability. However, the overall removal efficiency of *n*-alkanes by the bacterial consortium and rhamnolipid was higher than the control. Similar trend was observed for PAH and biomarkers (Chen et al., 2013). These results were more or less consistent with another study which also used rhamnolipids in combination with a pre-adapted bacterial consortium (Nikolopoulou et al., 2013a), and which were more pronounced when nutrients were added to the treatments. The average specific degradation rate was reported to be 23, 20, and 10 times higher than the control for *n*C_15_, *n*C_20_, and *n*C_25_, respectively. The rhamnolipid also stimulated the growth of hydrocarbon degraders within 5 days, which were able to utilize 50% of the crude oil saturated fraction. In addition, LMW PAHs and, notably, the biomarkers pristane and phytane were also significantly degraded in the presence of rhamnolipid (Nikolopoulou et al., 2013a). The origin of nutrients (i.e., organic lipophilic or water-soluble) that are added together with rhamnolipids to the treatments can further enhance the degradation of crude oil in seawater and sediment environments (McKew et al., 2007; Nikolopoulou and Kalogerakis, 2008; Nikolopoulou et al., 2013b).

Bioemulsifiers can also be used in oil spill response with promising results. A bioemulsifier exopolysaccharide produced by *Acinetobacter calcoaceticus*, called EPS_2003_, was shown to be effective in enhancing crude oil biodegradation in natural seawater microcosms (Cappello et al., 2012). The addition of EPS_2003_ to the microcosms not only enhanced hydrocarbon-degrading bacteria, including *Alcanivorax, Marinobacter, Oceanospirillum*, and *Pseudomonas*, but also caused a 2-fold faster biodegradation of the total oil compared to microcosms without the EPS (Cappello et al., 2012). All of these studies, however, focused entirely on the degradation rate of crude oil on a handful of selected oil-degrading bacteria without investigating the indigenous marine microbial community response as a whole. Understanding how natural microbial communities are affected by the crude oil with/without presence of biosurfactants is crucial for advancing the rationale for further research into their suitability for oil spill response. However, due to varying reasons, including current high costs of biosurfactants available on the market and logistics around field studies, there is a marked lack of reported studies investigating the effectiveness of biosurfactants as oil spill treating agents.

We are aware of only one study that compared a synthetic chemical dispersant and a biosurfactant. In that case, a surfactin produced by *Bacillus* sp. strain H2O-1 was compared to the synthetic dispersant Ultrasperse II (Couto et al., 2016). Surfactin enriched for hydrocarbonoclastic bacteria more so than the synthetic dispersant, but no difference in oil biodegradation between the two was observed. In more recent work, rhamnolipid from *P. aeruginosa* and a synthetic chemical dispersant were compared on the response of a natural bacterial community to crude oil (Nikolova et al., 2020). The rhamnolipid promoted higher diversity in oil-degrading bacteria than the synthetic dispersant, however, the crude oil was ultimately more biodegraded when synthetic dispersant was added to the oil, with the exception of the aromatic fraction of the oil. Notably, the synthetic dispersant resulted in a clear inhibition of *Cycloclasticus*—a genus comprising species of obligate oil-degrading bacteria which are recognized for using aromatic hydrocarbons as a preferred source of carbon and energy (Head et al., 2006).

Although biosurfactants may have a positive effect on the oil-degrading microbial community, it is necessary to advance their performance in dispersing crude oil. Developing novel types of biogenic surface-active compounds and more environmentally friendly technologies to combat large offshore oil spills is fertile ground for ongoing and future exploration. In this respect, research and development of biosurfactants for treating oil spills at sea has significantly intensified, particularly over the past 10 years due mainly to concerns over the enormous quantities of the synthetic chemical dispersant Corexit that were used during the Deepwater Horizon oil spill in the Gulf of Mexico. Much of this anew activity ensued through the Gulf of Mexico Research Initiative (GoMRI) and is discussed in the following section.

### Recent Trends in the Development of Bio-Based Dispersants to Combat Marine Oils Spills

Growing awareness among society regarding the environmental hazards associated with the use of chemical dispersants has led to increased interest toward the use of naturally-derived, biological dispersing products (i.e., biosurfactants) which are commonly associated with low toxicity, high biodegradability, better environmental compatibility, and are sustainably sourced compared to their counterparts (i.e., surfactants) that are produced via organo-chemical synthesis in a laboratory or industrial chemical plant (Desai and Banat, 1997). Over the past decades, numerous marine microorganisms, especially bacteria, have been identified that are able to degrade hydrocarbons by producing effective biosurfactants (Al-Wahaibi et al., 2014; Cai et al., 2014; Chandankere et al., 2014). However, the current knowledge on microbial biosurfactants has been limited to only a few compounds produced by a small number of bacteria and yeast species, such as *Pseudomonas, Bacillus, Candida*, and *Acinetobacter* (Ruggeri et al., 2009). These organisms, and their produced biosurfactants, have potential promise for application in offshore oil spill response, enhanced oil recovery, and soil washing treatment of petroleum-contaminated sites (Banat et al., 2010; De Almeida et al., 2016). However, it is important to find other promising biosurfactant-producing bacteria and/or yeast (and other fungi) in order to increase the variability of these biomolecules available for large-scale production and also to decrease the dependence on some of these microbial genera which have species of known human pathogens (e.g., *Pseudomonas aeruginosa, Candida*, and *Bacillus*). A highly promising source for discovering novel biosurfactant-producing microorganisms is the marine environment as it harbors an extensive and largely untapped microbial biodiversity, which has shown itself as a proven repository of powerful molecules currently used for pharmacological, food, and cosmetics applications (Kennedy et al., 2011; Gudiña et al., 2016; Perfumo et al., 2018).

The development of a new generation of dispersants that are as, or more, effective than commercial synthetic dispersants, cost efficient, and have minimal side effects when they come in contact with, or are ingested by, marine organisms and humans is a path that has gained traction since the Deepwater Horizon disaster. Through GoMRI in the U.S.A. as part of the Consortium for the Molecular Engineering of Dispersant Systems (C-MEDS), a number of projects have been underway aiming to develop bio-based dispersants, either from microorganisms or other natural sources, or using food-grade ingredients (e.g., silica, polyethylene glycol) that are common additives in food and medicine, and that can be obtained relatively cheaply by the ton. Whilst some of these C-MEDS projects utilize non-biomaterials, below we summarize some that use materials from biological sources so in keeping with the context of this review.

Led by scientists at Texas A&M Galveston as part of the Aggregation and Degradation of Dispersants and Oil by Microbial Exopolymers (ADDOMEx) group have shown that EPS produced by microorganisms (micro-algae and bacteria) is more efficient at oil dispersal than the synthetic chemical dispersant Corexit (Schwehr et al., 2018). In particular EPS with a higher protein-to-polysaccharide ratio resulted in higher enzymatic action and marine-oil-snow sedimentation efficiency, higher microbial diversity and cell abundance, and in more extensive biodegradation compared to oil treatments with Corexit, although the latter maintained a more stable emulsion of the oil droplets. Their findings also showed that the microbes in natural samples of seawater were more stressed when exposed to the crude oil or the oil together with Corexit, and in response they release more EPS that is higher in protein and carbohydrate/sugar content, but the EPS aggregates that form do not grow large enough to eventually sediment down. In the absence of Corexit, however, protein-rich EPS is formed, and which is significantly more efficient in purging the water column from the oil. Following this, the researchers are exploring ways to trigger the production of protein-rich EPS by natural communities of microorganisms in seawater during the event of another large oil spill.

Scientists from the University of Maryland and Tulane University have investigated the use of food-grade emulsifiers as substitutes for synthetic chemical dispersants. By examining the stability of emulsions of crude oil in seawater when in the presence of various food-grade emulsifiers, they found that lecithin (a cell membrane component) from soybean in combination with Tween 80 (emulsifier used in ice cream and other foods) were found to effectively disperse and produce more stable emulsions of crude oil than Corexit (Athas et al., 2014; Riehm et al., 2015). In another C-MEDS project, researchers are working on new classes of “green” dispersants made from naturally occurring inorganic and biomolecular materials. Using combinations of natural clay minerals and new carbon materials with synthetic polymer-based materials, new products are being evaluated to test for adhering strongly to the oil-water interface and stabilize oil droplets, which could prevent the formation of large slicks. C-MEDS researchers from the University of South Florida published research showing cactus mucilage dispersed crude oil more efficiently than synthetic dispersants, and notably requiring lower concentrations (Alcantar et al., 2015). In other work, biopolymers derived from cactus mucilage and chitosan show promise in synergistically working together with chemical dispersants, potentially helping to reduce the use of solvents that are typically intrinsic in synthetic chemical dispersant formulations.

Taking a different approach, C-MEDS researchers are also exploring new natural gelation agents in order to prevent oil slicks from spreading and, consequentially, reaching coastlines. Work led by Tulane University and collaborators used a gel-like matrix incorporated with Tween 80 and lecithin which resulted in improved stabilization of crude oil in seawater emulsions—more so over longer periods compared to traditional liquid dispersants (Owoseni et al., 2018). The gel-like formulation was designed to largely replace DOSS, the key surfactant component of synthetic chemical dispersants, with lecithin. The application of food-grade surfactants into a gel-like mesophase acts as a compact buoyant pod for improved delivery of the surfactants to sea surface oil slicks. It does this by way of remaining afloat where the oil is largely confined and avoiding the use of polypropylene glycol and the generation of volatile solvents in the atmosphere through aerial or ship-based spraying.

Researchers from the University of Texas at Austin investigated the potential use of nanoparticles as non-conventional dispersants and as tools to improve existing surfactant-based dispersants. Their work has led to the development of nanoparticles that are less toxic, more efficient oil spill treatments compared to synthetic chemical dispersants. By mixing hydrophilic nanoparticles (i.e., bare colloidal silica) with a weakly interacting zwitterionic surfactant (caprylamidopropyl betaine) to generate a high hydrophilic-lipophilic balance, the nanoparticles, and surfactant acted synergistically in forming finer emulsions with enhanced stability, particularly so in a seawater aqueous environment (Worthen et al., 2014). Caprylamidopropyl betaine (CAPB) is a surfactant that is formed using fatty acids from coconut or palm kernel oil and used in personal care products.

Scientists from Brown University and the University of Rhode Island studied the interactions of the obligate hydrocarbon-degrading bacterium *Alcanivorax borkumensis* with oil across oil-water interfaces that had varying amounts of the following different surfactants: CTAB (cetylytrimethylammonium bromide), lecithin (from soybean), SDS (sodium dodecyl sulfate), AOT (dioctyl sulfosuccinate sodium salt), and Tween 20, and they compared this to Corexit as the “gold” reference standard. The researchers recorded changes in the growth rate, lag time, and cell density of *A. borkumensis* at the oil-water interface containing low to high levels of these surfactants and found that not all of them aided this organism's degradation of the oil (Bookstaver et al., 2015). The food-grade surfactant, Tween 20, was found to work best by synergistically working with the organism, increased the surface area of oil droplets, and resulting in more bacterial growth and oil degradation. Conversely, the other surfactants inhibited the adherence of the bacterial cells to oil, limiting its biodegradation capacity. In conclusion, the authors recommended further investigation into the use of different surfactants, in particular Tween 20, to replace the current stockpile of synthetic chemical dispersants to treat future oil spills. In a similar study, researchers from the University of Houston compared the food-grade surfactant Tween 20 with several synthetic chemical dispersants to determine how they affect the adhesion of the hydrocarbon-degrading species *Marinobacter hydrocarbonoclasticus* to oil droplets (20–60 um), which is for some hydrocarbon-degrading bacteria an initial key step for biodegradation (Dewangan and Conrad, 2018). They found that increasing concentrations of all surfactants tested resulted in reduced adhesion of the cells to oil droplets, though electrostatic charge associated with some of the surfactants tested appeared to influence adhesion. Their results suggest that the choice of surfactant(s) in dispersant formulations should be accounted for with respect to how it affects bacterial adhesion to oil droplets and, hence, the biodegradation process.

In a study led by Tulane University in collaboration with Lappeenranta University of Technology, Finland, the stability of carboxymethylated chitosan nanoparticles cross-linked with either magnesium, calcium or strontium ions were studied under different pH and salinity in an effort to determine which could be used in oil spill treatment (Kalliola et al., 2016). The nanoparticles cross-linked with calcium ions, as well as when cross-linked with dodecane, were found to be most stable, showing potential for oil-spill treatment. In one other study, scientists from different institutions in Canada conducted work on the design of a lipopeptide biosurfactant produced by *Bacillus subtilis* N3-1P from fish waste-based peptone as a primary nutrient substrate for this bacterium (Zhu et al., 2020). The produced lipopeptide was evaluated as an ingredient together with DOSS, which is the key surfactant ingredient found in Corexit 9500. At a biodispersant ratio of 80/20 (v/v) of lipopeptide/DOSS, a high dispersion efficiency was achieved of 76.8% for Alaskan North Slope crude oil.

## Conclusion and Future Perspectives

Biosurfactants and their HMW/polymeric versions (bioemulsifiers) have gained high interest in recent years, due largely to consumer demand for natural ingredients and by companies in search of chemical ingredients conferring improved functional properties and that can be derived from sustainable sources. With respect to the Oil and Gas industry, the application of biosurfactants for MEOR and in dispersant formulations to treat oil spills are areas of significant interest, but not yet applied on an industrial scale. In the case of MEOR, despite the fact that it has been around for around 70 years, it has not been widely used by the industry, mainly because of a lack of multidisciplinary research to resolve many of the limitations or knowledge gaps that hinder its advancement. It is recognized that research to improve MEOR requires the discovery and isolation of new types of microbial strains, especially those that are active under anaerobic conditions and that can rapidly produce biomass or biosurfactants. One avenue could be in the genetic engineering of new bacterial strains to be made to be more efficient, such as for the *ex situ* production of useful biosurfactants or surface-active biopolymers for injection into oil reservoirs to allow the remaining oil to be more easily recoverable.

In the case of treating oil spills, chemical dispersants have been used for over 50 years and are the preferable treatment for marine oil spills. Many hard lessons have been learned from the Deepwater Horizon spill, one of which has spurred interest to search for alternative types of dispersants that have greater environmental compatibility. Dispersants that contain surfactants derived from biological sources (i.e., biosurfactants) are thus promising. We may have entered a new era in the development of a new generation of dispersants produced from biological sources (i.e., bio-dispersants). Whilst prevention of oil spills in the first place is paramount, more reliance on bio-based dispersants to treat oil spills at sea should help reduce the potential detrimental environmental impacts that synthetic chemical dispersants can cause. In particular, it will be important to select dispersants that speed up the rate and extent that spilled oil is biodegraded by oil-degrading populations of microorganisms.

## Author Contributions

CN sourced the information for the manuscript and together with TG, wrote the manuscript. All authors contributed to the article and approved the submitted version.

## Conflict of Interest

The authors declare that the research was conducted in the absence of any commercial or financial relationships that could be construed as a potential conflict of interest.

## Footnotes

^1^Cumulative annual growth rate.
